# When Organic Meets Solid: A Crystal-Clear Path to Better Batteries

**DOI:** 10.1021/acscentsci.6c00932

**Published:** 2026-06-09

**Authors:** Xiaodong Lin, Alexandru Vlad

**Affiliations:** † Institute of Condensed Matter and Nanosciences, 83415 Université Catholique de Louvain (UCLouvain), Louvain-la-Neuve B-1348, Belgium; ‡ WEL Research Institute, Wavre 1300, Belgium

## Abstract

A single-crystalline organic cathode shows how conductivity, crystallinity, and microstructure unlock high-capacity solid-state batteries.

## Graphical abstract

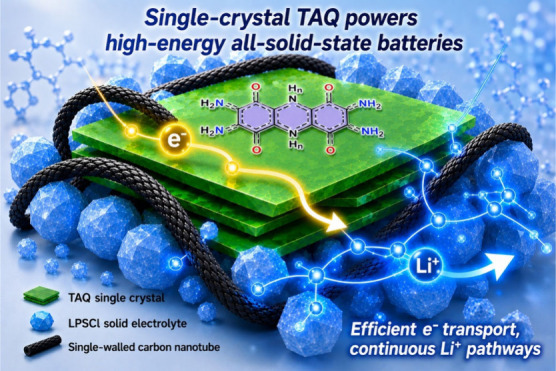

In a recent issue of *ACS Central Science*, Dincă and co-workers report a strikingly practical result for a field that often struggles to bridge molecular design with device-level constraints: a single-crystalline, semiconductive, layered organic cathode that delivers high capacity as a positive electrode in all-solid-state batteries (ASSBs).^1^ The headline is not simply “another organic cathode,” but rather a rare convergence of features: crystallinity, intrinsic electronic transport, and electrode-level optimization, all directly targeting long-standing bottlenecks at solid–solid interfaces.

The headline is not simply “another organic cathode,” but rather a rare convergence of features: crystallinity, intrinsic electronic transport, and electrode-level optimization, all directly targeting long-standing bottlenecks at solid–solid interfaces.

Why does this matter? ASSBs promise improved safety and can enable the use of lithium metal, yet they routinely pay a penalty at the composite cathode: limited ionic percolation, incomplete utilization of the active phase, and interfacial degradation that is difficult to diagnose without the buffering effects of liquid electrolytes.^2,3^ Organic electrode materials are attractive for sustainability and high theoretical capacities, but in solid-state architectures they face an additional handicap: low electronic conductivity often demands high carbon fractions, which consume volume that would otherwise be allocated to the solid electrolyte and thereby disrupt ionic pathways.^4,5^ The present work tackles this challenge directly using bis-tetraaminobenzoquinone (TAQ), a layered, hydrogen-bonded fused aromatic material that is intrinsically semiconductive (reported electronic conductivity >10^–5^ S cm^–1^) and can access multielectron redox.^1^

A central strength of the study is its explicit “electrode engineering” logic, which is frequently underdeveloped in organic cathode reports. Rather than focusing on a single composite, the authors map performance across a controlled series of TAQ–solid electrolyte–carbon ratios and reveal a clear and intuitive trade-off: increasing active-material loading beyond an optimum can reduce capacity because the solid electrolyte fraction drops below the percolation threshold required for continuous Li^+^ transport.^1^ In other words, the limiting factor is not always the redox chemistry; it is often the geometry and continuity of ion pathways in a rigid, multiphase electrode. This microstructure-to-performance relationship is summarized schematically in Figure 1, which highlights the percolation window and the composition optimum that translates intrinsic TAQ capacity into usable solid-state capacity.

**Figure 1:**
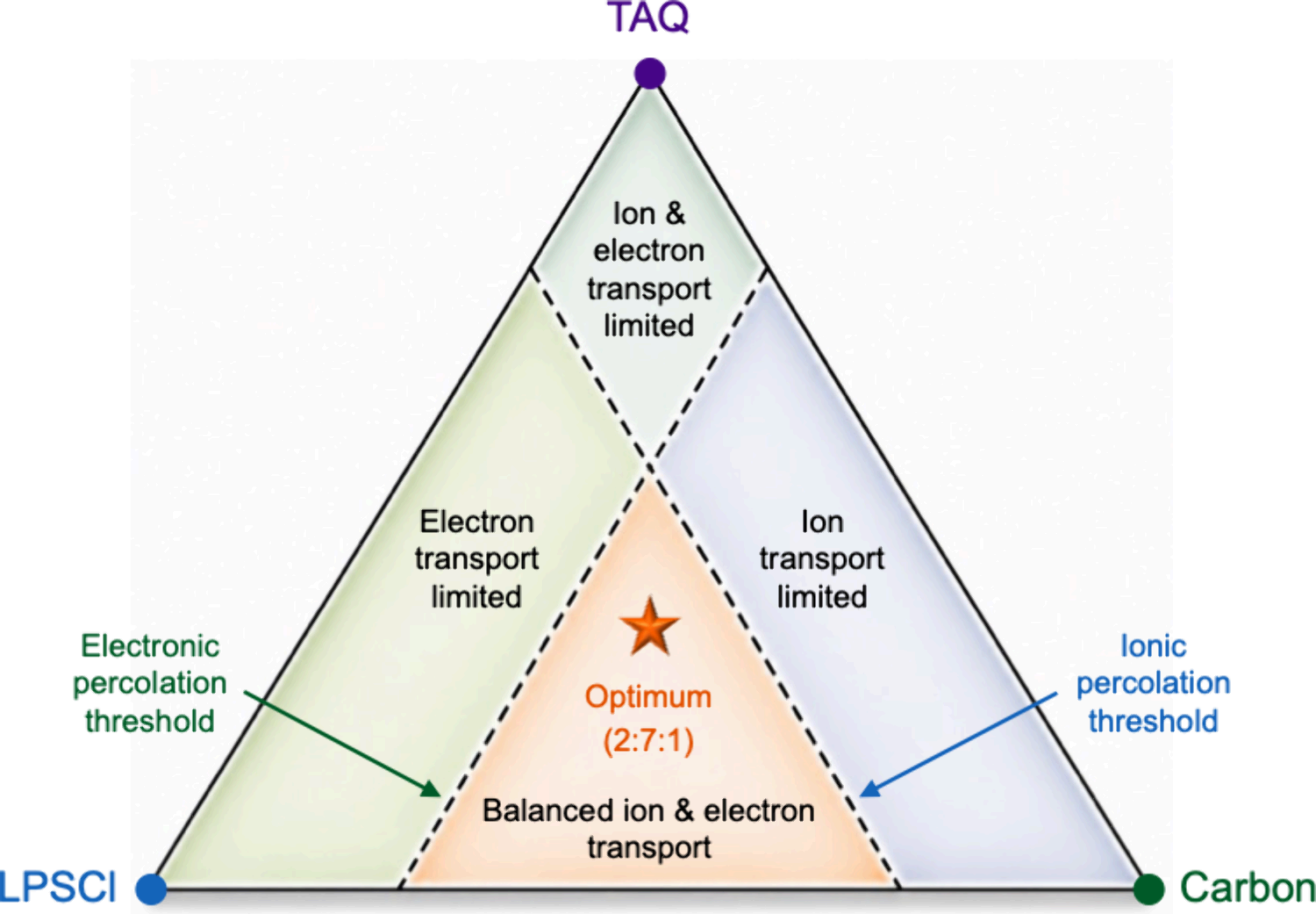
Qualitative ternary composition map (schematic; not to scale) illustrating percolation-limited regimes in TAQ/LPSCl/carbon composite cathodes and the representative optimum composition (2:7:1).

The authors map performance across a controlled series of TAQ–solid electrolyte–carbon ratios and reveal a clear and intuitive trade-off: increasing active-material loading beyond an optimum can reduce capacity because the solid electrolyte fraction drops below the percolation threshold required for continuous Li^+^ transport.

The authors then connect this compositional optimization to transport in a way that is unusually explicit for organic cathodes in ASSBs. *Ex situ* impedance spectroscopy shows simplified behavior compared with many liquid cells, consistent with reduced parasitic processes under the studied conditions.^1,3^ From these analyses, Li^+^ diffusion coefficients are extracted and shown to span orders of magnitude across the composite compositions, while the measured electronic conductivity varies comparatively little—an important practical message: once a cathode is “conductive enough,” solid-state performance becomes primarily ion-transport-limited.^1^

One of the most conceptually interesting observations is the state-of-charge dependence of Li^+^ transport. Using *in situ* PEIS, the authors report a volcano-shaped behavior: diffusion is suppressed at both Li-poor and Li-rich end points but peaks at intermediate occupancy, an intuitive signature of coupled effects from structural evolution and site availability.

But probably one of the most conceptually interesting observations is the state-of-charge dependence of Li^+^ transport. Using *in situ* PEIS, the authors report a volcano-shaped behavior (a conceptual representation of this volcano-like transport landscape is provided in Figure 2): diffusion is suppressed at both Li-poor and Li-rich end points but peaks at intermediate occupancy, an intuitive signature of coupled effects from structural evolution and site availability. Such transport landscapes can be masked in liquid electrolytes, where continuous wetting and rapid ion supply can hide local bottlenecks. Here, the solid-state configuration becomes an advantage: it exposes the coupling between crystal structure, occupancy, and kinetics in a more direct way.

**Figure 2:**
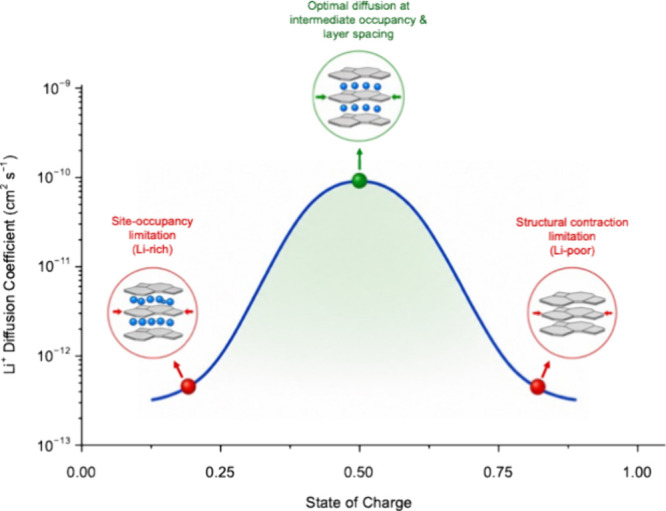
Conceptual volcano-shaped dependence of Li^+^ diffusion on state of charge in layered TAQ, illustrating site-occupancy limitation at Li-rich states and structural contraction (reduced interlayer spacing) limitation at Li-depleted states. Qualitative schematic; not to scale.

Another important message is that crystal quality matters disproportionately in ASSBs. The authors compare high-crystallinity and low-crystallinity TAQ and find large penalties in capacity and stability for the nanocrystalline material. This is not merely a synthetic nuance. Grain boundaries, heterogeneity, and mechanical fragility can translate directly into interfacial resistance and loss of contact in rigid solid-state assemblies, effects that are amplified when ionic percolation is already marginal.^2,3^ For organic cathodes, where community intuition sometimes leans toward “amorphous is enough,” this work argues for a more nuanced rule: when electronic transport and mechanical integrity are limiting, highly ordered morphologies can be enabling, even if they complicate processing.

The study also offers two practical “levers” that could be broadly generalizable. First, compositing TAQ with single-walled carbon nanotubes strengthens electronic percolation by replacing point contacts with extended conductive pathways, an architecture that can reduce the carbon penalty without sacrificing connectivity. Second, operating at elevated temperature improves ion transport and mitigates polarization, clarifying which bottlenecks originate in the organic redox host and which are imposed by ion transport through the composite.

Where does this leave the field? The results are encouraging because they demonstrate that organic cathodes can be competitive in sulfide-based ASSBs at practical pressures and room temperature, reaching high active-material-level capacity and stable cycling.^1,2^ Yet the work also sharpens the roadmap toward cell-level relevance. First, active-material-level metrics are valuable, but competitiveness at the cell level will depend on increasing areal loading while maintaining percolation and minimizing dead volume, an engineering challenge closely connected to processing routes compatible with sulfide electrolytes.^7^ Second, the stability window and interphase chemistry of sulfide electrolytes remain central; the paper suggests reduced side reactions, but deeper mechanistic understanding of cathode–electrolyte interphases for organic solids is still less mature than for inorganic oxide cathodes.^3,6,9^ Finally, manufacturing matters: the authors’ composites are hand-prepared, and translating these insights to scalable processing (dry coating, solvent-assisted routes compatible with sulfides, or templated architectures) is likely where much of the next improvement will be won.^7,8^

The results are encouraging because they demonstrate that organic cathodes can be competitive in sulfide-based ASSBs at practical pressures and room temperature, reaching high active-material-level capacity and stable cycling.

Overall, Dincă and his team provide more than a strong data set, they provide a design map: build intrinsic electronic transport into the organic cathode, preserve order and robustness, and treat the composite microstructure as a first-class variable.^1^ If organic electrodes are to play a serious role in next-generation solid-state energy storage, this is exactly the kind of work that will make them legible and credible to the broader ASSB community.^2,8^
